# PARTNERING TO ADDRESS ADULT MALTREATMENT: HARD-EARNED LESSONS AND WISDOM GAINED BY APPLIED RESEARCHERS

**DOI:** 10.1093/geroni/igad104.1572

**Published:** 2023-12-21

**Authors:** Courtney Reynolds, Samantha Tuft, Farida Ejaz

**Affiliations:** Benjamin Rose Institute on Aging, Cleveland, Ohio, United States; Benjamin Rose Institute on Aging, Cleveland, Ohio, United States; Benjamin Rose Institute on Aging, Cleveland, Ohio, United States

## Abstract

We focus on three projects in which applied researchers collaborated with community-based and government partners to address adult maltreatment. In our first study with Texas, we partnered with a large healthcare system and Adult Protective Services (APS). Two APS workers served as consultants to clinicians and 529 patients were reported to APS. Researchers struggled with quantifying case notes on the consultations, pointing to the importance of creating a systematic method to document case notes for future studies. In the second grant, the same partners evaluated a case management intervention to prevent self-neglect among at-risk, adult primary care patients. A total of 477 randomly selected participants completed a baseline interview. Lessons learned included revising language in how to discuss self-neglect, particularly in consent forms and becoming more flexible in where to conduct interviews. Other challenges encountered included the time and effort it took to develop data use agreements that allowed us to access APS administrative data. Our third study with Oklahoma APS was conducted in the midst of a pandemic, requiring us to change study protocols from home to telephone-based interviews. Despite these challenges, we interviewed 188 clients over the phone. Drawing on the experiences across all three projects, we will discuss challenges – from contracting to IT issues, to recruitment and data collection – and how each one has presented an opportunity for growth and wisdom that can be applied to future research activities.

